# Acceptance of E-Mental Health Services for Different Application Purposes Among Psychotherapists in Clinical Training in Germany and Switzerland: Secondary Analysis of a Cross-Sectional Survey

**DOI:** 10.3389/fdgth.2022.840869

**Published:** 2022-02-28

**Authors:** Pia Braun, Marie Drüge, Severin Hennemann, Felix Jan Nitsch, Robert Staeck, Jennifer Apolinário-Hagen

**Affiliations:** ^1^Faculty of Medicine, Institute for Occupational, Social and Environmental Medicine, Centre for Health and Society, Heinrich Heine University Düsseldorf, Düsseldorf, Germany; ^2^Department of Clinical Psychology/Psychotherapy Research, Institute of Psychology, University of Zurich, Zurich, Switzerland; ^3^Department of Clinical Psychology, Psychotherapy and Experimental Psychopathology, Institute of Psychology, University of Mainz, Mainz, Germany; ^4^Marketing Area, INSEAD, Fontainebleau, France; ^5^Paris Brain Institute (ICM), INSERM U 1127, CNRS UMR 7225, Sorbonne Université, Paris, France

**Keywords:** acceptance, eHealth, eMental health, psychotherapists, telemedicine, unified theory of acceptance and use of technology

## Abstract

**Background:**

Despite solid evidence supporting the efficacy of electronic mental health (EMH) services, their acceptance among psychotherapists is limited and uptake rates remain low. However, the acceptance of different EMH services has yet barely been examined in future generations of psychotherapists in a differentiated manner. The aims of this study were (1) to elaborate the intention to use various EMH services for different application purposes and (2) to determine predictors of EMH service acceptance among psychotherapists in clinical training (PiT).

**Materials and Methods:**

Our paper is based on a secondary data analysis of a cross-sectional survey. Respondents were recruited via recognized educational institutions for psychotherapy within Germany and the German-speaking part of Switzerland between June and July of 2020. The survey contained items on the intention to use different EMH services (i.e., guided and unguided programs, virtual reality, psychotherapy by telephone and videoconference) for various application purposes (i.e., prevention, treatment addition, treatment substitute, aftercare). Potential predictors of EMH service acceptance (e.g., barriers and advantages) were examined based on an extension of the Unified Theory of Acceptance and Use of Technology (UTAUT).

**Results:**

Most of the *n* = 216 respondents were female (88.4%) and located in Germany (72.2%). General acceptance of EMH was moderate (*M* = 3.4, *SD* = 1.12, range 1–5), while acceptance of psychotherapy via videoconference was highest (*M* = 3.7, *SD* = 1.15) and acceptance of unguided programs was lowest (*M* = 2.55, *SD* = 1.14). There was an interaction effect of EMH service and application purpose (η^2^ = 0.21). Barriers and advantages both had a uniform influence on EMH service acceptance (Pr > 0.999), while impersonality, legal concerns, concerns about therapeutic alliance, simplified information provision, simplified contact maintenance, time flexibility, and geographic flexibility were significant predictors (all *p* < 0.05). Results showed that the extended UTAUT model was the best fitting model to predict EMH service acceptance (Pr > 0.999).

**Conclusions:**

The intention to use different EMH services varied between application purposes among PiT. To increase acceptance of EMH services and reduce misconceptions, we identified predictors that should be addressed in future acceptance-facilitating interventions when educating PiT.

## Introduction

During the ongoing COVID-19 pandemic, common mental health disorders (CMDs) such as depression, post-traumatic stress disorder (PTSD) or anxiety disorders increased tremendously across the globe (1–4). High prevalence rates for CMDs can oftentimes be linked to perceived uncertainty, fear and social isolation measures that come along with this global health crisis (5–7). To offer quick, safe and location-independent help, the World Health Organization (8) has recommended to ensure access to psychosocial support services through digital systems. Consequently, the need for easily accessible, effective and flexible services as alternatives or additions to traditional mental health treatment to support vulnerable populations became even more evident during the COVID-19 pandemic (9).

Electronic mental health (EMH) services are usually internet-delivered services that have proven to be effective in trials on the prevention and treatment of CMDs (10–13), for instance in reducing symptoms of PTSD (14), anxiety (13, 15), depression (16), panic disorder and social anxiety disorder (17). EMH services have been advancing into routine care in developed countries even before the outbreak of the COVID-19 pandemic, as they can complement and improve health care systems (10). Principally, EMH interventions have several advantages over face-to-face interventions such as time flexibility and greater accessibility because they are location-independent and thereby could conquer structural barriers (18, 19). Additionally, EMH services offer a low-threshold, anonymous option for individuals who are afraid of stigmatization (19). Other drivers include perceived acceleration of the treatment process and outcome, simplified contact maintenance (20), improved adherence, health literacy and disease management (21).

Despite these advantages and well documented efficacy of EMH interventions (22–24) the dissemination remains low in many countries at an earlier stage of digital health implementation into healthcare such as Switzerland or Germany (25–27). Efficient implementation of EMH services depends on the utilization and acceptance by potential users and health experts. According to the Unified Theory of Acceptance and Use of Technology (UTAUT), acceptance can be operationalized as the intention to use technology and serves as a direct predictor of the actual usage (9, 28). Thus, low uptake rates can be explained by EMH acceptance being low to moderate among patients (25, 29–31) and health professionals (32, 33). The UTAUT model emerged from eight different acceptance models and was initially developed for the work context (28), but has been successfully validated and adapted to digital health care (9). It is the most frequently used model providing a theoretical framework for potential factors that predict acceptance, including performance expectancy, effort expectancy about the ease to use technical services, social influence by stakeholders and facilitating conditions, as e.g., the extent to which organizational and technical structures support the use of services (34). Performance expectancy is supposed to be the strongest predictor (9), representing beliefs of relative advantage or usefulness of the technical service. Beyond these well documented UTAUT factors, other predictors of EMH acceptance that have been suggested by research, are personal experience with EMH and electronic health (eHealth) literacy (i.e., the ability to find, evaluate, and utilize internet-based health information) (35, 36), knowledge about EMH services (30, 37) and the perceived evidence base on the effectiveness of EMH services (38).

In general, EMH acceptance seems to be even lower among health professionals such as psychotherapists compared to patients (39, 40). Barriers that are perceived by psychotherapists are diverse, including insufficient information (21) concerns about the technology itself (e.g., data security and privacy), lack of clear ethical guidelines and concerns about relational aspects (20, 37, 41, 42). Additionally, a comprehensive legal and regulatory framework for psychotherapists, along with reimbursement schemes, is often lacking even though awareness at the policy level is increasing (43). As health experts are often the primary source of health information or treatment recommendation (44), they supposedly have a large influence on patients' attitude formation and thus on the implementation of EMH services (35). Hence, research should focus on understanding both acceptability and attitudes as determinants of behavioral intentions to use and actual utilization of health experts as negative attitudes can result in slow dissemination or poorer uptake of EMH services (45, 46).

EMH is an umbrella term that includes a wide range of electronic services (e.g., self-help, psychoeducational information, virtual reality, psychotherapy via videoconference, counselling, etc.) which are applied for different purposes, such as for prevention or treatment of CMDs (47). About a decade ago, Eichenberg and Ott (44) could show that most EMH services were used for treatment (71%), 19.1% for prevention and only 9% for rehabilitation purposes. Meanwhile, digital health applications (medical apps) for mental health such as selfapy (48), velibra (49) or deprexis (50) have been integrated into routine care in Germany in fall 2020 and are now used along the entire patient journey (51). Medical apps are guided or unguided programs which are self-directed mobile phone- or web-based programs that entail information and a fixed number of modules or exercises for (mental) health problems (12, 48, 50). Oftentimes, the basis of guided medical apps is internet-based cognitive behavioral therapy (iCBT) which involves the user following a written electronic treatment program, together with receiving synchronous or asynchronous support from a therapist via e-mail, texts or calls (52). This therapeutic approach has been shown to be effective in reducing anxiety disorders (53), depressive symptoms (23), suicidal ideation (54) or insomnia (55). In Germany, medical apps can be prescribed by physicians for self-help purposes, aftercare or relapse prevention (38, 56). In Switzerland, medical apps are similarly used, expanding their traditional health care system (57). For self-help purposes, unguided programs are most often used as they offer a possibility to monitor and better understand perceived symptoms and help users to take actions on their own to improve their mental health (12). For aftercare and rehabilitation purposes, professionally guided programs have been predominantly implemented, with health experts supporting clients in health promotion by providing some sort of synchronous or asynchronous interaction or feedback in addition to unguided services (12). Nevertheless, reducing EMH to medical apps would fall short as there are several more ways to use EMH. For instance, there is psychotherapy via videoconference or telephone which is most often used as an alternative treatment delivery service, either as an addition to or substitute for face-to-face-therapy (58). It has been shown to be an effective and timely treatment option for depression and anxiety disorders, especially for patients living in rural areas (58). However, the evidence base of the efficacy of psychotherapy via videoconference or telephone is still scarce and researchers have only started to investigate the efficacy of this EMH service with the outbreak of the COVID-19 pandemic (59–61). Virtual reality (VR) is another EMH service that has been used for diagnostic purposes (62), for prevention (63), and the treatment of a range of CMDs in clinical settings (62, 64). For instance, VR therapy has been shown to be a valuable treatment for social anxiety (65), panic disorder (66) or PTSD (67).

Clearly, EMH services are characterized by great heterogeneity of applied methods, target groups, desired objectives and scientific evidence (68). However, EMH acceptance has yet barely been examined in a differentiated manner with regard to specific areas of application. Thus, general conclusions about EMH acceptance fall short. Instead, it is necessary to assess the intention to use various EMH services for different application purposes to get an extensive picture. Therefore, the research aim of this study was (1) to directly compare the acceptance of psychotherapy via telephone, psychotherapy via videoconference, VR, unguided and guided programs among psychotherapists in clinical training (PiT) for different application purposes, including prevention, treatment substitute and treatment addition in acute care as well as aftercare. Additionally, factors that potentially predict EMH acceptance have most often been assessed in general and not for distinct EMH services. Thus, another aim of this study was (2) to apply an extended UTAUT model to exploit which predictors best determine EMH service acceptance. We chose PiT as our study population because they will shape the future healthcare system. In Germany and Switzerland, PiT already hold a university degree in either psychology or medicine and are now in their postgraduate clinical training which is required to obtain the state-approved permission to practice psychotherapy. Even though the advancing digital transformation of healthcare has already started to shape the professional routines and careers of PiT, their perspective has rarely been included in research.

## Materials and Methods

### Study Design

This is an exploratory secondary analysis based on data derived from a cross-sectional survey-study that was carried out by a research team of the University of Zurich in summer 2020. For the primary analysis the acceptance and perceived barriers of EMH were calculated as an average of five different EMH services (psychotherapy via telephone, psychotherapy via videoconference, VR, unguided and guided programs) among PiT. The current acceptance scores of EMH services were compared to pre-COVID-19 acceptance scores, which were assessed retrospectively. Results will be reported elsewhere in full length^1^. Participants were recruited between June and July of 2020 via recognized educational institutions for psychotherapy within Germany and the German-speaking part of Switzerland. Recruitment was administered solely via e-mail, asking the post-gradual educational institutions to forward the link to the survey to PiT. Thereby, PiT were directed to the survey, which was conducted online and completely anonymous. The survey contained 50 questions and mean processing time was 19.1 min (*SD* = 5.9). As an incentive, participants could take part in a raffle of book vouchers worth 50 euros. Institutions were contacted again if they did not answer the request after 2 weeks. In total, 29 institutions in Switzerland and 232 institutions in Germany were contacted. Since only a few institutions gave feedback on forwarding the questionnaire, no statement can be made about the response rate on an institutional level. In total, the questionnaire was opened 692 times, with 228 PiT completing the survey which results in a dropout rate of 68.7 %. We could not control for multiple clicking, thus the dropout rate might appear higher than it actually is 0.12 participants were excluded from analyses as they had not started the practical part of their postgraduate clinical training yet. After written consultation with the President of the Ethics Committee of the University of Zurich on 3 March 2020 and the checklist to self assess ethical safety, no further approval of the ethics committee was necessary to garantuee the ethical safety of the study.

### Measures

#### Sociodemographic Characteristics

The survey contained items on sociodemographic data covering age categorized in eight subgroups (20–24 to 55–59, each category including 5 years) to preserve anonymity of respondents, sex, education, country of education (Switzerland or Germany) and theoretical orientation (i.e., behavioral therapy, depth psychology or psychoanalysis, systemic therapy, humanistic therapy). Following sociodemographic questions, the survey continued with a definition of EMH (47) and each EMH service (68).

#### Primary Outcome

Acceptance was operationalized according to UTAUT (28). Consequently, acceptance was assessed using three items: “I could imagine including the following EMH services in my work”, “I intend to try out the following EMH service in my work within the next year”, and “How high is your intention to use the following EMH service in your work ever?”. The first two items were rated on a 5-point Likert scale ranging from (1) *totally disagree* to (5) *totally agree*. The third item was rated on a scale ranging from 0 to 100 and adapted from Elfeddali et al. (69) to measure the intention strength. For statistical analyses, the third item was converted into a 5-point Likert scale and a mean score of all three items was calculated for EMH acceptance.

#### Secondary Outcomes

Acceptance of different EMH services for various application fields was operationalized as the intention to use psychotherapy via telephone, psychotherapy via videoconference, VR and unguided as well as guided programs for prevention, therapy substitute in acute care, therapy addition in acute care and aftercare (e.g., “Which EMH services would you use for prevention?”). All items were rated on 5-point Likert scales ranging from (1) *totally disagree* to (5) *totally agree*, with higher scores indicating elevated acceptance. The UTAUT predictors performance expectancy (e.g., “The following EMH service would be a useful extension to existing treatment measures”), effort expectancy (e.g., “I would find the following EMH service easy to use and to understand”), social influence (e.g., “My colleagues would approve the use of the following EMH service”) and facilitating conditions (e.g., “I have the necessary preconditions for using the following EMH service”) were measured each with two items that were partly adapted from previous studies (28, 33). Answers were rated on a 5-point Likert scale ranging from (1) *totally disagree* to (5) *totally agree*. Barriers (i.e., data insecurity, impersonality, irresponsibility, legal concerns, concerns about therapeutic alliance) and advantages (i.e., time flexibility, simplified information provision, geographic flexibility, and simplified contact maintenance) were assessed as other possible predictors of acceptance and also based on previous studies (70–73). Additionally, the survey included three items on the knowledge about EMH services that were adapted from Hennemann et al. and Ebert et al. (e.g., “I know what I can expect when using virtual reality as a therapeutic tool”) (28, 33). Answers were rated on a 5-point scale ranging from (1) *totally disagree* to (5) *totally agree*. The item on EMH experience in their role as healthcare provider (e.g., “In percentage, how much do you already use the following EMH services in your therapeutic work?”) was adapted from previous studies (33). The item on evidence assessment of EMH services (e.g., “How would you rate the scientific evidence base of the following EMH services?”) was self-constructed. All items that we used for our analyses can be found in the Supplementary Materials, including the English translation.

### Statistics

Data were analyzed using IBM SPSS Statistics 26 and R (Version 4.0.0). To answer the question whether the acceptance of EMH services varies between application purposes, we used as a statistical model a 2-factor within-subject (repeated measure) ANOVA with the factors EMH services (five steps: psychotherapy via telephone, psychotherapy via videoconference, VR treatment, unguided programs, guided programs) and application purposes (four steps: prevention, treatment substitute, treatment addition, aftercare) and EMH acceptance as dependent variable. The model included both main effects (EMH services and application purposes), as well as their interaction (EMH services x application purposes).

To identify how different barriers to the acceptance of EMH services might differentially affect EMH service types, we adopted a two-step approach. First, we identified an appropriate model of the relation of the barriers to the different EMH services in terms of general acceptance. Specifically, we considered three candidate linear mixed-effects models in our model set. All models included a main effect of EMH service type and a random subject intercept. The random subject intercept was included as acceptance was assessed multiple times, that is once per EMH service for each participant (i.e., as a repeated measure). This is a standard procedure to account for within-subject correlation of measures (e.g., see (74), p. 29). The first model (A1) additionally included a main effect of all five barriers each (data insecurity, impersonality, irresponsibility, legal concerns, concerns about therapeutic alliance), as well as pair-wise interaction terms of each barrier and EMH service type. Hence, this model represented a differential relationship of barriers to EMH service acceptance depending on the type of service. The second model (A2) dropped the interaction terms, hence representing a uniform influence of the barriers on EMH acceptance. The third model (A3) dropped the main effect terms of the five barriers, representing no influence of the barriers on EMH acceptance. Our criterion of model comparison was based on Akaike Information criterion (AIC) weights (75), which express the probability that a model is the best in the model set conditional on the data. Second, we inspected the regression coefficients of the best fitting model specifically for the five barriers to gain insights on which barriers had a significant influence on EMH acceptance.

We followed an equivalent procedure to better understand the influence of advantages of EMH services. Again, we firstly identified an appropriate descriptive model, considering three candidate linear mixed-effects models in our model set. All models included a main effect of EMH service type and a random subject intercept. The first model (B1) additionally included a main effect of all four advantages each (simplified information provision, time flexibility, geographic flexibility, simplified contact maintenance), as well as pair-wise interaction terms of each advantage and EMH service type. Hence, this model represented that the relationship of advantages to EMH service acceptance depended on the type of service. The second model (B2) dropped the interaction terms, hence representing a uniform influence of the advantages on EMH acceptance. The third model (B3) dropped the main effect terms of the four advantages, representing no influence of the advantages on EMH acceptance. Again, we inspected the regression coefficients of our best fitting model specifically for the four advantages, to gain insights on which of them had a significant influence on EMH acceptance.

Lastly, we aimed to put the different pieces of our data modelling together within the UTAUT framework. Specifically, we wanted to test if adding possible influences of barriers and advantages (depending on the analyses above) presented a meaningful extension to the classic UTAUT predictors and simple comparison model featuring only demographic predictors (age, gender). All models included a main effect of EMH service type, age, gender, and a random subject intercept. In addition, model C1 included the UTAUT predictors (performance expectancy, effort expectancy, social influence, and facilitating conditions), the barriers and advantages, as well as knowledge about, experience with and subjective assessment of the scientific evidence base of different EMH services as they have been shown to have an influence on EMH acceptance. Model C2 only additionally included the UTAUT predictors, while model C3 did not include additional predictors. Again, our criterion of model comparison was based on Akaike Information criterion (AIC) weights.

## Results

### Sociodemographic Characteristics

**Figure 1:**
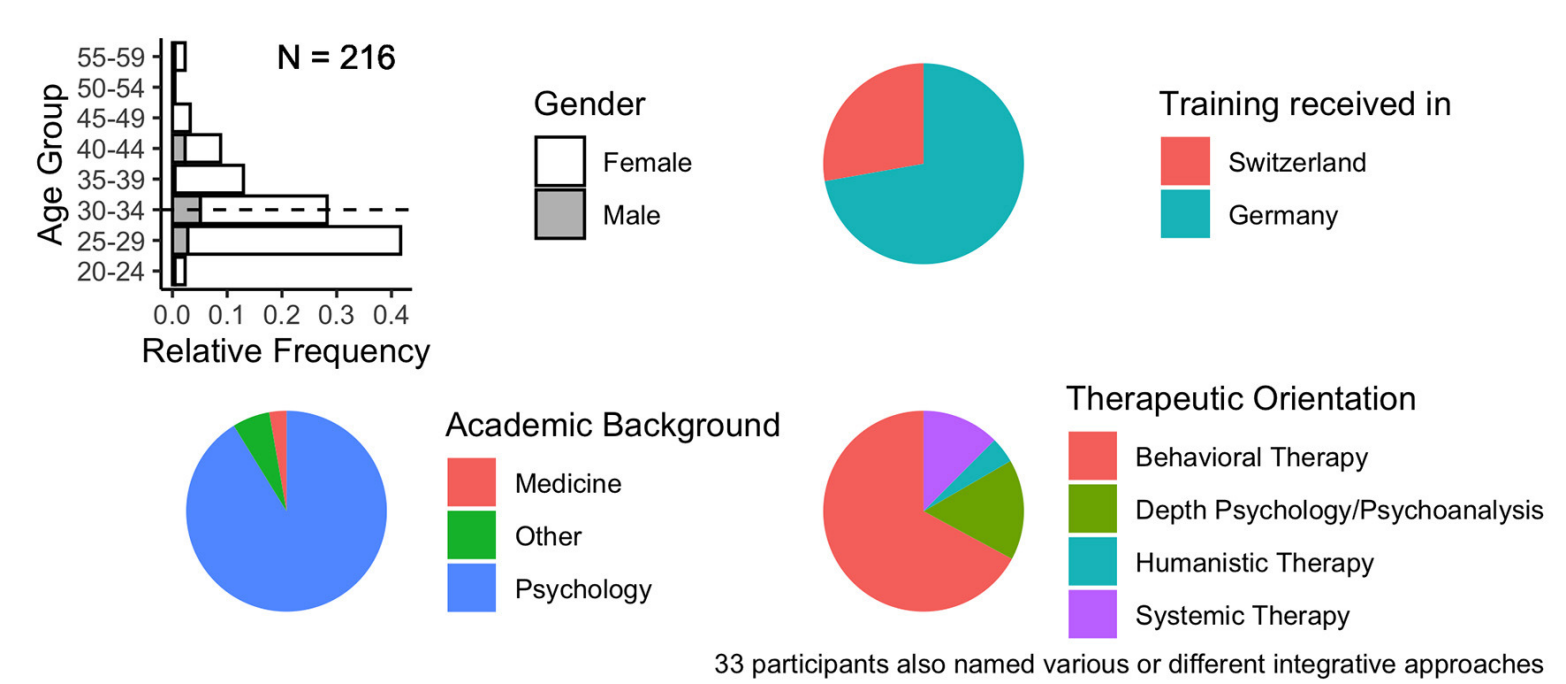
Participant Demographics.

Figure 1 provides a summary of key sociodemographic characteristics. The sample size was *n* = 216 participants, with *n* = 60 participants who trained in Switzerland (27.8%) and *n* = 156 in Germany (72.2%). Most of them were female (88.4%) and between 25 and 39 years old (85.2%). *N* = 197 respondents studied psychology (91.2%) and *n* = 6 medicine (2.8%) before starting with their clinical training to become a psychotherapist and *n* = 13 indicated completing other degrees (6%). Regarding the theoretical orientation, 67.1% stated that they are trained in behavioral therapy (cognitive/cognitive-behavioral), 16.2% in depth psychology or psychoanalysis, 12.5% in systemic therapy, and 4.2% in humanistic therapy. *N* = 33 participants named various or different integrative approaches (15.3%).

### Acceptance of EMH

Based on prior research (33, 70) the mean score of EMH acceptance was categorized as low (1–2.34), moderate (2.35–3.67), or high (3.68–5). In general, results revealed that acceptance of EMH was moderate (*M* = 3.4, *SD* = 1.12), while acceptance of psychotherapy via videoconference was highest (*M* = 3.7, *SD* = 1.15) and acceptance of unguided programs was lowest (*M* = 2.55, *SD* = 1.14). Table 1 gives an overview.

**Table 1:** Means and standard deviations of EMH service acceptance.

	***M***	***SD***
Acceptance of psychotherapy via telephone	3.36	1.21
Acceptance of psychotherapy via videoconference	3.7	1.15
Acceptance of VR treatment	2.7	1.1
Acceptance of unguided programs	2.55	1.14
Acceptance of guided programs	2.88	1.14
General acceptance of EMH	3.4	1.12

Among respondents, general perceived personal knowledge about EMH was moderate (*M* = 3.64, *SD* = 0.86), while psychotherapy via videoconference was most well-known (*M* = 4.34, *SD* = 0.72). Practical experience with EMH was generally low, as participants stated using EMH services in only one out of ten therapeutic cases (*M* = 10.37, *SD* = 10, range 0–100%) between the onset of the COVID-19 pandemic and the time of data collection (June-July 2021). However, there were considerable differences between EMH services and high variance scores within psychotherapy via telephone and videoconference. Psychotherapy via videoconference (*M* = 26.55, *SD* = 28.80) and via telephone (*M* = 23.05, *SD* = 25.07) was used in about one out of four therapeutic cases. Participants indicated serving only *M* = 1.34% (*SD* = 2.2) of their patients with VR. Lastly, PiT recommended unguided EMH programs to only *M* = 3.38% (*SD* = 10.50) of their patients, while they stated that they have accompanied *M* = 4.19% (*SD* = 12.95) of their patients with guided programs.

### Acceptance of EMH Services for Different Application Purposes

**Figure 2:**
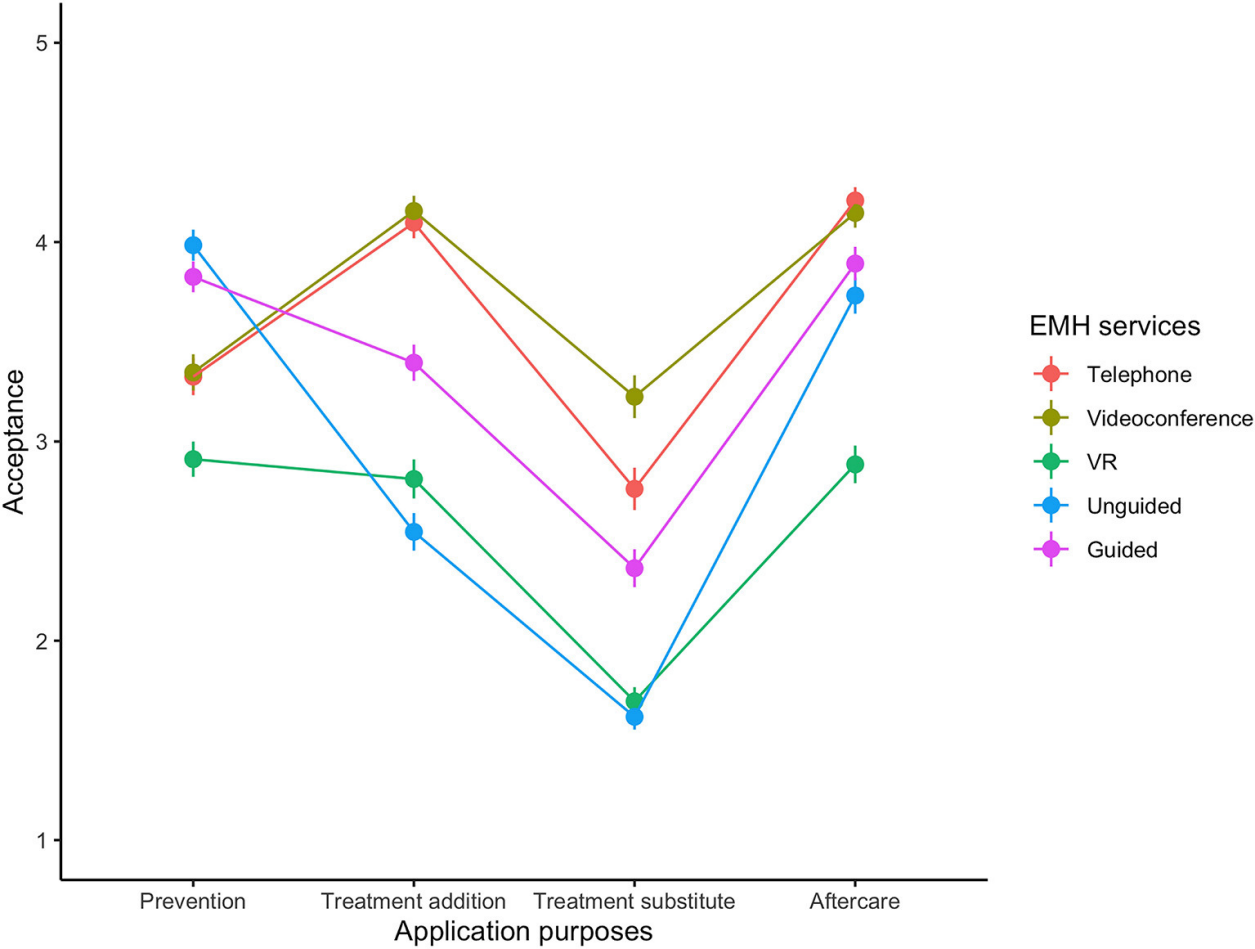
Mean Acceptance Scores for different EMH Services across Application Purposes.

Figure 2 provides an overview of the key results. Mauchly tests for sphericity revealed relevant violations (all *p* < 0.001) wherefore we report Greenhouse-Geisser corrected statistics. Our results confirmed the expected heterogeneity in the acceptance of different types of EMH services depending on their intended application purpose. Specifically, we found an interaction effect of EMH service and application purpose (F(6.229, 1283.088) = 111.497, *p* < 0.001, η^2^ =0.21). Post-hoc tests showed that, on average, over all application purposes, psychotherapy via videoconference was the most accepted EMH service (all *p_bonferroni* < 0.001). Further, EMH services were comparatively less accepted as a treatment substitute in acute care than for other application purposes (all *p_bonferroni* < 0.001). Interestingly, unguided and guided programs were specifically well accepted in preventive care (more so than all other services, all *p_bonferroni* < 0.059). VR was comparatively less accepted across all application purposes (all *p_bonferroni* < 0.001).

Beyond comparative statements, we used one-sample, one-sided Wilcoxon signed-rank tests against test value of 3 (neutral) to test which EMH services for which application purposes were seen as a valuable addition to the therapy catalogue on absolute scale. This was the case in 11 of 20 combinations. Specifically, results show that EMH services, except VR, are seen as useful for prevention and aftercare whereas they are not accepted as treatment substitution. Table 2 summarizes the results.

**Table 2:** V-statistics of EMH service acceptance for different application purposes.

	**V**	***p***
Prevention—psychotherapy via telephone	10,323.000	**<0.001**
Prevention—psychotherapy via videoconference	10,859.000	**<0.001**
Prevention—VR treatment	5,731.500	0.870
Prevention—Unguided EMH programs	13,156.500	**<0.001**
Prevention—Guided EMH programs	11,408.500	**<0.001**
Treatment addition—psychotherapy via telephone	16,788.000	**<0.001**
Treatment addition—psychotherapy via videoconference	18,368.500	**<0.001**
Treatment addition—VR treatment	5,649.500	0.962
Treatment addition—unguided EMH programs	3,791.500	1.000
Treatment addition—guided EMH programs	8,176.500	**<0.001**
Treatment substitute—psychotherapy via telephone	7,089.000	0.991
Treatment substitute—psychotherapy via videoconference	10,608.000	0.084
Treatment substitute—VR treatment	1,059.500	1.000
Treatment substitute—unguided EMH programs	441.000	1.000
Treatment substitute—guided EMH programs	3,050.000	1.000
Aftercare—psychotherapy via telephone	19,372.000	**<0.001**
Aftercare—psychotherapy via videoconference	18,352.000	**<0.001**
Aftercare—VR treatment	5,176.500	0.958
Aftercare—unguided EMH programs	12,061.000	**<0.001**
Aftercare—guided EMH programs	12,968.000	**<0.001**

*Wilcoxon signed-rank test*.

*For all tests, the alternative hypothesis specifies that the median is >3*.

*Values indicated in bold are significant*.

### Determinants of EMH Service Acceptance

#### Influence of Barriers on the Acceptance of EMH Services

To identify how different barriers to the acceptance of EMH services might differentially affect EMH service types, we considered three candidate linear mixed-effects models in our model set. All models included a main effect of EMH service type and a random subject intercept. The first model (A1) additionally included a main effect of all five barriers each, as well as pair-wise interaction terms of each barrier and EMH service type. The second model (A2) represented a uniform influence of the barriers on EMH acceptance, while the third model (A3) represented no influence of the barriers on general EMH acceptance. Our model comparison unequivocally favored model A2 (Pr > 0.999), suggesting that barriers had a uniform influence on general EMH acceptance.

An inspection of the regression coefficients of model A2 revealed that impersonality, therapeutic alliance, and legal concerns were significant predictors of EMH service acceptance (in decreasing order of regression weight – predictors were assessed on a common scale; see Table 3).

**Table 3:** Estimates of barriers to the acceptance of EMH services.

	**EMH service acceptance**	
**Predictors**	**Estimates**	***p***
Constant	4.93 (0.14)	**<0.001**
EMH service: videoconference	0.17 (0.08)	**0.043**
EMH service: VR treatment	−0.43 (0.08)	**<0.001**
EMH service: unguided	−0.22 (0.09)	**0.010**
EMH service: guided	−0.28 (0.08)	**0.001**
Data Insecurity	−0.03 (0.03)	0.308
Impersonality	−0.24 (0.03)	**<0.001**
Irresponsibility	−0.05 (0.03)	0.150
Legal Concerns	−0.07 (0.03)	**0.027**
Concerns about Therapeutic Alliance	−0.16 (0.04)	**<0.001**

*N CASE 209*.

*Observations 991*.

*Marginal R2 / Conditional R2 0.298 / 0.557*.

*Values indicated in bold are significant*.

#### Influence of Advantages on the Acceptance of EMH Services

An equivalent procedure was followed to better understand the influence of advantages of EMH services. The first model (B1) included a main effect of all four advantages each, as well as pair-wise interaction terms of each advantage and EMH service type. The second model (B2) represented a uniform influence of the advantages on EMH acceptance. The third model (B3) represented no influence of the advantages on EMH acceptance. Similar to our result for the barriers, our model comparison unequivocally favored model B2 (Pr > 0.999), suggesting that advantages had a uniform influence on general EMH acceptance.

Inspecting the regression coefficients of Model B2, we found that all four, that is simplified information provision, simplified contact maintenance, time flexibility, and geographic flexibility were significant predictors of EMH service acceptance (in decreasing order of regression weight; see Table 4).

**Table 4:** Estimates of drivers to the acceptance of EMH services.

	**EMH service acceptance**	
**Predictors**	**Estimates**	***p***
Constant	0.96 (0.17)	**<0.001**
EMH service: videoconference	0.26 (0.08)	**0.001**
EMH service: VR treatment	−0.51 (0.10)	**<0.001**
EMH service: unguided	−0.77 (0.09)	**<0.001**
EMH service: guided	−0.69 (0.09)	**<0.001**
Simplified information provision	0.27 (0.03)	**<0.001**
Time flexibility	0.14 (0.03)	**<0.001**
Geographic flexibility	0.09 (0.04)	**0.012**
Simplified contact maintenance	0.18 (0.03)	**<0.001**

*N CASE 209*.

*Observations 991*.

*Marginal R2 / conditional R2 0.361 / 0.578*.

*Values indicated in bold are significant*.

#### Advanced UTAUT Model

**Figure 3:**
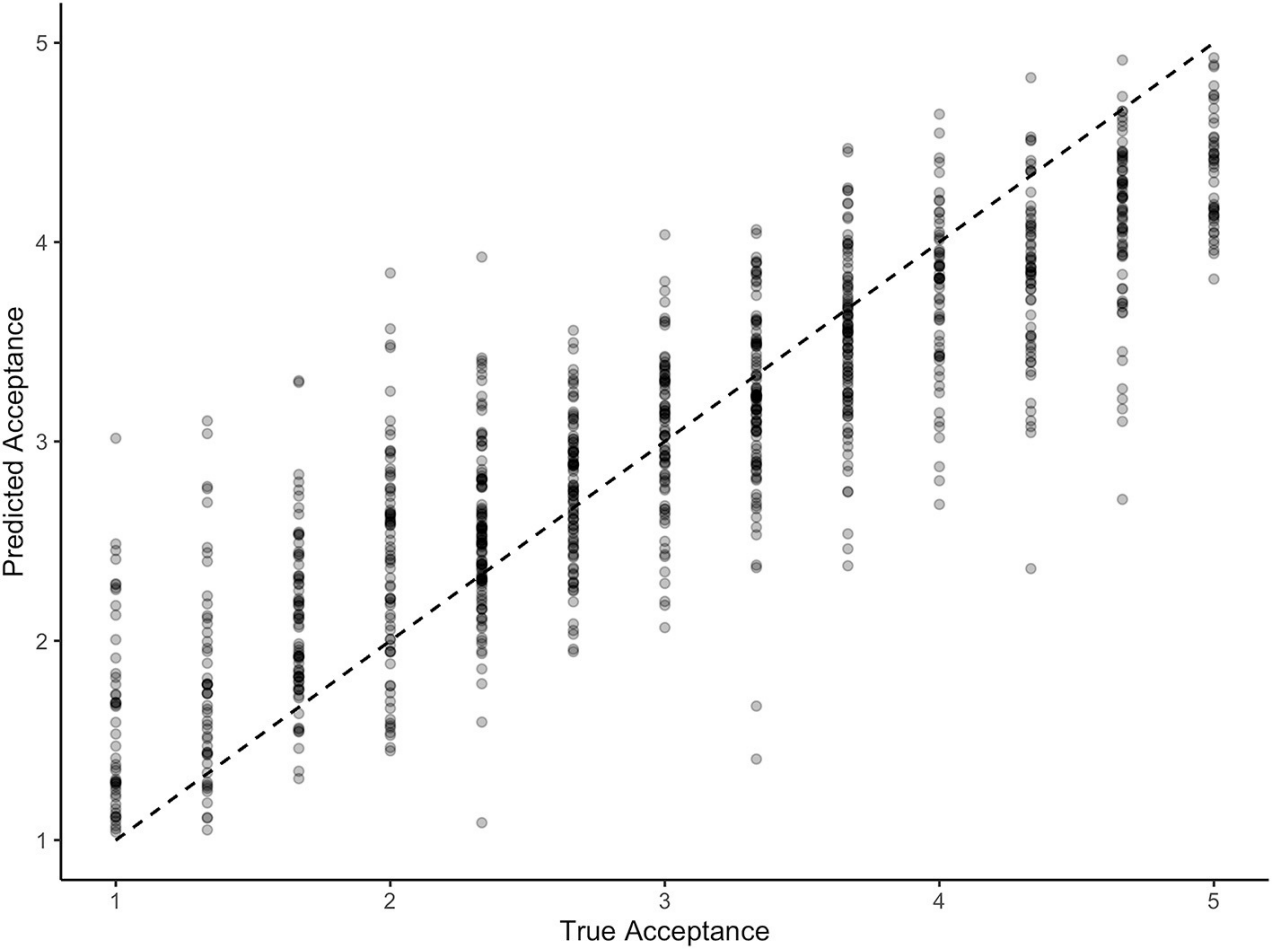
Predictive Performance of the Advanced UTAUT Model.

Lastly, we wanted to test if adding the uniform influences of barriers and advantages (as suggested by the analyses above) presented a meaningful extension to the classic UTAUT predictors and a simple comparison model. Our results confirmed that the extended UTAUT model (C1) which included the UTAUT predictors, the barriers and advantages, as well as knowledge about, experience with and subjective assessment of the scientific evidence base of different EMH services was the best given the model set and the data (Pr >0.999), explaining 74% of variance. Table 5 shows the regression coefficients, while Figure 3 visualizes the predictive performance of model C1.

**Table 5:** Estimates of EMH service acceptance determinants (advanced UTAUT model).

	**EMH service acceptance**	
**Predictors**	**Estimates**	***p***
Constant	0.29 (0.36)	0.414
Age: 25–29	−0.19 (0.25)	0.449
Age: 30–34	−0.15 (0.25)	0.563
Age: 35–39	−0.11 (0.27)	0.672
Age: 40–44	−0.08 (0.28)	0.770
Age: 45–49	−0.17 (0.32)	0.603
Age: 50-54	−0.47 (0.60)	0.428
Age: 55–59	−0.10 (0.35)	0.785
Gender: male	−0.16 (0.12)	0.189
EMH service: videoconference	−0.02 (0.07)	0.808
EMH service: VR treatment	−0.20 (0.10)	0.059
EMH service: unguided	−0.06 (0.09)	0.521
EMH service: guided	−0.25 (0.09)	**0.004**
Experience with EMH services	0.01 (0.00)	**<0.001**
Knowledge about EMH services	0.04 (0.03)	0.181
Evidence assessment of EMH services	0.01 (0.00)	**<0.001**
Data Insecurity	0.01 (0.02)	0.733
Impersonality	−0.06 (0.03)	**0.038**
Irresponsibility	−0.01 (0.03)	0.655
Legal concerns	−0.00 (0.02)	0.851
Concerns about therapeutic alliance	−0.10 (0.03)	**<0.001**
Simplified information provision	0.09 (0.02)	**<0.001**
Time flexibility	0.07 (0.03)	**0.005**
Geographic flexibility	−0.02 (0.03)	0.432
Simplified contact maintenance	0.07 (0.03)	**0.009**
UTAUT: performance expectancy	0.36 (0.04)	**<0.001**
UTAUT: social influence	0.19 (0.04)	**<0.001**
UTAUT: facilitating conditions	0.01 (0.03)	0.654
UTAUT: effort expectancy	0.08 (0.04)	0.078

*N CASE 209*.

*Observations 991*.

*Marginal R2 / conditional R2 0.584 / 0.738*.

*Values indicated in bold are significant*.

## Discussion

The present study aimed at exploring the acceptance of various EMH services among German-speaking PiT shortly after the global outbreak of the COVID-19 pandemic that has forced changes in the provision of psychological support around the world for the prevention, treatment and aftercare of CMDs.

Accordingly, there was an urgent need for valid and flexible EMH services as alternatives or additions to traditional mental health in-person measures in spring 2020. In our paper, we primarily focused on the intention to use unguided and guided EMH programs, psychotherapy via telephone, psychotherapy via videoconference and VR treatment as EMH services and prevention, therapy addition, therapy substitute and aftercare as application purposes among PiT during the first wave of the COVID-19 pandemic in Germany and the German-speaking part of Switzerland. Based on an adapted UTAUT model (28, 70), we included performance expectancy, effort expectancy, facilitating conditions and social influence as potential predictors of EMH service acceptance as well as barriers (i.e., data insecurity, impersonality, liability, legal concerns, and concerns about therapeutic alliance), advantages (i.e., time flexibility, simplified information provision, geographic flexibility, and simplified contact maintenance), EMH knowledge, experience with EMH and subjective assessment of the scientific evidence base of different EMH services.

### Main Findings and Comparisons With Prior Work

#### Acceptance of EMH Services for Different Application Purposes

First of all, the acceptance of EMH was overall moderate among PiT. In general, acceptance of psychotherapy via videoconference was highest, while acceptance of unguided programs was lowest. This is in line with Gerlinger et al. (38), who could show that healthcare providers are, in principle, receptive to the possibilities of such unguided programs. However, from the healthcare providers' point of view, the preconditions for a successful integration into the healthcare system are not yet fulfilled. Even though a recent survey among the twenty biggest social health insurance companies in Germany shows an upward trend regarding prescription rates of medical apps, numbers are still relatively low with projected 45.000 prescriptions (76). In comparison, according to the Scientific Institute of the National Health Insurance Schemes and the Federal Association of Company Health Insurance Funds (AOK) about 685 million finished medicinal products were prescribed in 2020 (56). Nevertheless, when looking at acceptance rates across different application purposes, our results show that guided and unguided EMH programs were specifically well accepted in preventive care, even more so than all other services including synchronous interactions between the patient and therapist via videoconference or telephone. In fact, prior research has demonstrated that unguided and guided EMH programs such as medical apps are perceived as being helpful for the promotion of patient empowerment by physicians and psychotherapists (38) which has been shown to be related to health status in the general population (77). Concerning prevention and health promotion purposes, there seems to be a greater emphasis on self-help activities (e.g., help for self-help), which could be well supported by structured self-help programs, such as stress management trainings, mental health apps and early interventions. Moreover, primary prevention does not fall into the therapeutic field and does not require a trained psychotherapist to guide these kinds of nontherapeutic interventions. Additionally, our results show that EMH services, except VR, are also well accepted for aftercare purposes. At least for health experts, our results seem to be in line with prior research. For instance, Hennemann et al. (33) could show that acceptance of online aftercare for work-related stress was moderate among health professionals of various professional groups including physicians and psychologists in inpatient rehabilitation facilities. Similar to preventive care, EMH services seem to be promising tools to overcome barriers to the utilization of traditional aftercare, such as limited local accessibility, temporal incongruity with work and private life, concerns about anonymity or stigmatization (78–80). Thus, to support patients in health promotion and self-efficacy in their rehabilitation process, health experts tend to accept EMH services.

Furthermore, we identified the highest acceptance of psychotherapy using videoconference software for complementary treatment purposes, as well as similarly high acceptance ratings for therapeutic interactions via telephone. In contrast, EMH services were comparatively less accepted as a treatment substitute in acute care than for other application purposes. Particularly, as a treatment substitute psychotherapy via videoconference was accepted most, while all other EMH services were rated relatively low. Potentially, PiT prefer having more visible control of the acute treatment process including the therapeutic alliance and feel more comfortable with direct synchronous communication, including the interpretation of verbal and nonverbal signals. Interestingly, the evidence base of the effectiveness of psychotherapy via videoconference or telephone is a still a growing research area (60, 61, 81, 82) and there is considerably more evidence on the treatment effectiveness and acceptance of structured EMH self-help programs such as minimally guided iCBT which also forms the basis of some medical apps for mental health (48). From the perspective of potential clients, individuals seem to generally prefer these therapist-guided internet interventions such as iCBT over videoconferencing and unguided internet interventions when they have to choose between different EMH services (25) as well as blended delivery modes combining online or telephone contact with face-to-face psychotherapeutic sessions (83). At least for acute treatment purposes, we found contrasting results for PiT which could be explained with comparatively low practical experience with EMH services and self-reported little knowledge about EMH services. Additionally, within guided EMH programs we did not differentiate between whether oneself as a PiT is guiding the client through the EMH program or another, additional therapist which could be of interest for future research.

Moreover, our results indicate that VR was comparatively less accepted across all application purposes in the sense that VR treatment did not score highest in any purpose. Again, this result can be explained by respondents indicating having almost no experience with VR, while at the same time, having at least modest experience with psychotherapy via videoconference, which was applied in about one out of four therapeutic cases on average. Lacking knowledge about possible advantages and disadvantages of VR might have resulted in a low willingness for future use as past research has shown a link between usage experience and acceptance (28–30). Additionally, the acceptance of VR may be reduced due to technical requirements and may further depend on its yet restricted application options especially in the context of PTSD and anxiety disorders, such as specific phobia (e.g., exposure to feared stimuli via systematic desensitization).

In line with other research, our results clearly show that EMH acceptance should be assessed distinctly as it varies between EMH services, target groups and application purposes. For instance, research by Apolinário-Hagen et al. (45) revealed that self-help books, health websites and face-to-face counselling were perceived as more useful than web-based counselling and therapies within the general population. Hennemann et al. (33) found limited acceptance of EMH interventions among health professionals of inpatient treatment, while results revealed moderate acceptance of online aftercare for work-related stress. Among licensed psychotherapists in Austria, Schuster et al. (84) could show a preference for blended (face-to-face plus web-based) interventions over web-based interventions to treat CMDs. Varying results from study to study can be linked to distinct study populations, different framing including varying application purposes and other time periods of data assessment. Additionally, a lack of shared terminology limits comparability between studies (85). Furthermore, despite these evident differences, EMH is often still assessed very broadly which leads to less meaningful results. Hence, future research should put emphasis on these differences when assessing acceptance, elicit possible explanations and agree on used terminology.

#### Determinants of EMH Service Acceptance

As potential advantages that influence the acceptance of EMH services, we identified simplified information provision, simplified contact maintenance, time flexibility, and geographic flexibility. Concerning perceived barriers, we found that impersonality, legal concerns, and therapeutic alliance were significant predictors of EMH service acceptance. Comparing different predictor models of the intention to use EMH services among PiT, the extended UTAUT model fitted our data best (model C1). Overall, our findings correspond to other research targeting the views and experiences of psychotherapists. Among European psychotherapists having mainly positive experiences with online consultations during the COVID-19 pandemic, De Witte et al. (43) reported several barriers that might hinder implementation, such as data security issues or concerns about relational aspects, for instance impersonality and fostering a therapeutic alliance. In a study by Sander et al. (86), German professionals reported having little experience or knowledge about internet-delivered interventions and the most frequently anticipated barriers were too severe symptoms of patients, the feared neglect of face-to-face contacts and insufficient technical equipment. The most frequently mentioned potential benefits were an optimized treatment structure and patient empowerment. Schuster et al. (84) found similar advantages of EMH services to be of importance, such as time and geographic flexibility, simplified information provision, patient empowerment but also discretion and the suitability for young patients. To further increase acceptance of and trust in EMH services, Gerlinger et al. (38) emphasize the need for verified evidence on the effectiveness, data security and interoperability of EMH services. Furthermore, the additional workload for health care providers should be transparently available before they use or prescribe EMH services, such as mental health apps.

In summary, EMH acceptance of PiT may be explained according to the UTAUT model when coupled with their perceptions of barriers and drivers as well as their practical experience as healthcare providers with EMH, knowledge about EMH and their perception of the scientific evidence base of EMH services. Even though the UTAUT model has recently been successfully validated and adapted to digital health care (9), our results show that it is necessary to extend this model and adapt it to the context of PiT given the complex nature of EMH acceptance and its determinants. In short, we did not assess all factors that could potentially influence EMH acceptance and focused on those that we perceived as being most important for PiT, knowing that there might still be missing factors that could be relevant. Congruently, Ammenwerth (87) pointed out that technology acceptance depends on multiple factors that have yet been overlooked, such as emotional, socio-organizational, cultural or workflow aspects. Thus, future research is needed to examine additional factors and strongest predictors to gain a deeper understanding of the intention to use different EMH services, while differentiating between target groups. This would help to design acceptance-facilitating interventions (AFIs) to educate PiT about different EMH services concerning applying them for prevention, treatment or aftercare purposes.

### Limitations

While this study contributes to the understanding of the acceptance of different EMH services for various application purposes and its determinants, it also has some limitations that should be considered. First, we must consider the time point of assessment. Data were gathered during the first months of the outbreak of the COVID-19 pandemic which could explain higher acceptance rates compared to older studies (32, 33, 45). The given circumstances have accelerated the use of remote services and forced psychotherapists to rethink about digital alternatives to treat patients. Additionally, the online survey included a description of structural benefits of psychotherapy via telephone or videoconference, especially in extraordinary conditions such as the COVID-19 crisis, which could have positively influenced acceptance scores for these two EMH services. At the same time, general acceptance rates could also be lower compared to newer studies as experience with EMH was still relatively low among respondents and EMH experience has been shown to be positively related to technology acceptance (28, 35, 88). Even if we consider the early stage of implementation of EMH services in Germany and Switzerland (38, 89, 90), healthcare experts have gained experience with digital medicine during the COVID-19 pandemic, the intention to use EMH services might increase concurrently.

In addition, the gender ratio was not balanced as more female than male psychotherapists in clinical training participated in our study which might have influenced our results. Moreover, the response rate was rather low, as on average less than one respondent per institution completed the survey. Age and gender were no predictors of acceptance in the advanced UTAUT model, which is likely due to the selection bias with few male participants and little variation in age. Female psychotherapists in some European countries like Germany have been shown to be more likely to endorse and provide digital psychotherapy during the first weeks of the COVID-19 outbreak in Europe, especially by those who were more concerned about an infection with COVID-19 (60). However, in our study we did not control for nontherapeutic reasons for providing digital psychotherapy, such as concerns regarding an infection.

Furthermore, the present study only focused on acceptance and fell short in the question of how behavioral intention and actual use behavior might be linked. Even though UTAUT describes behavioral intention as a direct predictor of the actual uptake (28), potential users do not always follow their intentions (“intention-behavior gap”, (91)). Thus, we agree with Philippi et al. (9) that future research should focus on the relationship between the intention to use different EMH services and use behavior (92) and investigate whether identified predictors of EMH acceptance could potentially influence actual uptake rates.

Lastly, the operationalization of technology acceptance was slightly different to other studies focusing on acceptance toward digital interventions, thus comparability is limited. Even though we based our assessment of behavioral intention on the frequently used UTAUT, individual adaptations of the UTAUT questionnaire and the number of items can differ between studies. For instance, acceptance is sometimes operationalized with four items (32, 93) or two items (33) that are rated on a 5-point scale ranging from (1) *does not apply at all* to (5) *applies completely*. Apolinário-Hagen et al. (45) only used one item by assessing intentions to use EMH services with an abbreviated version of the procedure applied by Klein and Cook (94), asking participants how likely they would use 10 different conventional and EMH services in case of emotional problems on a 5-point rating scale ranging from (0) *very unlikely* to (4) *very likely*. In our study, we used three items to assess behavioral intentions, including two items that were also used by Hennemann et al. (33) and one item asking psychotherapists in clinical training for their intention to use different EMH services in their work ever (range 0–100) that was adapted from Elfeddali et al. (69) to measure intention strength.

### Practical Implications

To expand the uptake of EMH, there is a need to focus on increasing psychotherapists' acceptance of EMH services as they play a crucial part in patients' attitude formation and thus on the implementation of EMH services (35). Our results provide evidence of the need to focus on informing prospect psychotherapists about advantages of various EMH services when applied in different contexts such as prevention and aftercare, but also on how potential barriers such as data security or legal concerns could be overcome. Confirmatory, a study by Humer et al (61) revealed that several psychotherapists in Austria wished for more information on data protection and security. Even before the COVID-19 pandemic, lack of personal contact, data protection and security were already seen as most important disadvantages of online interventions to prevent common mental health disorders by stakeholders such as psychotherapists, policymakers and potential users in Germany, Switzerland, Austria and Spain (95). Thus, these aspects of EMH services should be addressed in training and further education of psychotherapists. Additionally, a clear regulatory framework is needed to reduce legal concerns of psychotherapists. Countries in an earlier stage of digital health implementation into healthcare, such as Switzerland or Germany, could learn from countries that are more advanced in the implementation of EMH services such as the Netherlands or the United Kingdom (27). As a starting point, van Daele et al. (96) have recently formulated an association with the European Federation of Psychologists' Associations (EFPA) general guidelines for mental health workers, health services, regulatory agencies as well as developers to promote the implementation of evidence-based EMH services. The strong need for training and further education also becomes visible in a recent study by De Witte et al. (43), in which participants were asked whether they received any form of training on online consultations about EMH. Results revealed that only 11% of the sample received a form of training, however, only half of these training programs were specific to EMH and lasted just <4 h in every second case. In accordance, Gerlinger et al. (38) indicate that mental health workers do not feel well informed about possible benefits and risks of EMH services, while only few have already gained practical experience with EMH services (97, 98).

To address misconceptions and knowledge gaps through information provision, AFIs have been found to be an established tool in educating individuals about novel treatment options such as EMH services and in increasing their acceptance (30, 32, 45, 99). For instance, Baumeister et al. (32) could show that an AFI such as receiving a short video of blended therapy can increase performance expectancy, effort expectancy, facilitating conditions and overall acceptance toward blended therapy. In the future, similar AFIs could be integrated into the curricula of postgraduate training programs and continuous professional education to increase knowledge about our identified drivers (i.e., simplified information provision, simplified contact maintenance, time flexibility, and geographic flexibility) as well as barriers (i.e., impersonality, legal concerns, and therapeutic alliance) to the acceptance of EMH services. By making EMH an integral part of the education, PiT could gain valuable experience in integrating EMH services into their therapeutic work with patients.

Furthermore, PiT with varying theoretical backgrounds might need different education. For instance, unguided EMH programs such as mental health apps are most often based on cognitive behavioral therapy, which could lead to the assumption that psychotherapists with a background in cognitive behavioral therapy might be more open to use such EMH services (40, 100). In line with this assumption, Baumeister et al. (32) pointed out that particularly psychodynamic oriented psychotherapists could profit from AFIs as they initially seem to be rather skeptical about unguided EMH programs. Furthermore, There are already several studies that have identified associations between theoretical orientation (e.g., psychodynamic, cognitive behavioral, and others) and attitudes toward the use of EMH services (40, 100, 101), however findings are comparatively inconsistent, thus to deduce practical implications future research in this area is needed.

### Conclusions

This study is one of few to examine the acceptance of different EMH services (i.e., psychotherapy via videoconference, psychotherapy via telephone, VR, unguided and guided programs) across varying application purposes (i.e., prevention, treatment substitute, treatment addition, aftercare) from the perspective of PiT. We could show that acceptance for several EMH services differed for application purposes among PiT. The results showed that acceptance of EMH services was best predicted with an extension of the UTAUT model, including barriers (i.e., data insecurity, impersonality, liability, legal concerns, and concerns about therapeutic alliance), advantages (i.e., time flexibility, simplified information provision, geographic flexibility, and simplified contact maintenance), EMH experience, EMH knowledge, and EMH evidence assessment. As the use of EMH services will most probably increase in the next years because they offer quick and location-independent help for the prevention, treatment and aftercare of CMDs, our results highlight the need to distinctly inform PiT about different EMH services and their possible application areas. At the same time, our results provide support for stakeholders that are planning and designing training for PiT by highlighting factors that should be addressed if the goal is to increase EMH acceptance.

## Data Availability Statement

The original contributions presented in the study are included in the article/Supplementary Material, further inquiries can be directed to the corresponding author.

## Ethics Statement

After written consultation with the President of the Ethics Committee of the University of Zurich on 3 March 2020 and the checklist to self assess ethical safety, no further approval of the ethics committee was necessary to garantuee the ethical safety of the study involving human participants. The participants provided their written informed consent to participate in this study.

## Author Contributions

PB: conceptualization, project administration, and writing—original draft preparation. MD: conceptualization, supervision, methodology, and writing—reviewing and editing. SH: writing—reviewing and editing. RS: conceptualization, methodology, investigation, software, and data curation. FN: formal analysis, visualization, and writing—reviewing and editing. JA-H: supervision, validation, and writing—reviewing and editing. All authors have read, revised, and approved the final manuscript.

## Conflict of Interest

PB received payments from the Heinrich Heine University Dusseldorf for a lecture about electronic mental health services. PB also works for the Rehappy GmbH, which developed a digital health application for the rehabilitation of stroke patients. SH received payments from psychotherapy training institutes in the context of electronic mental health topics. The remaining authors declare that the research was conducted in the absence of any commercial or financial relationships that could be construed as a potential conflict of interest.

## Publisher's Note

All claims expressed in this article are solely those of the authors and do not necessarily represent those of their affiliated organizations, or those of the publisher, the editors and the reviewers. Any product that may be evaluated in this article, or claim that may be made by its manufacturer, is not guaranteed or endorsed by the publisher.

## Footnotes

^1^Staeck, R., Drüge, M., Albisser, S., and Watzke, B. (submitted). Acceptance of E-mental health interventions and its determinants among psychotherapists-in-training during the first phase of COVID-19.
