# Supplementary material for: Acceptance of E-Mental Health Services for Different Application Purposes Among Psychotherapists in Clinical Training in Germany and Switzerland: Secondary Analysis of a Cross-Sectional Survey
Source: Front Digit Health. 2022 Feb 28;4:840869. doi: 10.3389/fdgth.2022.840869 (PMC8918841; doi:10.3389/fdgth.2022.840869)
Supplement: Supplementary file 1 [file Data_Sheet_1.PDF]

test205612 → qnr2 19.08.2020, 12:56

Seite 01

IN01

Sehr geehrte/r Versuchsteilnehmer/in

Vielen Dank, dass Sie an der nachfolgenden Befragung zum Thema „**Akzeptanz von E-Health bei Psychotherapeut*innen in Ausbildung**“ teilnehmen, die wir im Rahmen zweier Masterarbeiten an der Universität Zürich (am Lehrstuhl Klinische Psychologie mit Schwerpunkt Psychotherapieforschung) durchführen. Hierbei soll unter anderem untersucht werden, ob sich die Akzeptanz bezüglich unterschiedlicher Anwendungsgebiete von E-Mental-Health unterscheidet.

Der Fragebogen wendet sich ausschliesslich an **Psychotherapeut*innen in Ausbildung**.

Der Fragebogen kann in ca. 20-30 Minuten ausgefüllt werden. Versuchen Sie zu jeder Frage eine Antwort zu finden und denken Sie daran: **es gibt hierbei kein "richtig" oder "falsch"**. Ihre persönliche Meinung zählt! Sie können die Befragung jederzeit abbrechen, ohne das Nachteile für Sie entstehen.

IN02

Ihre Teilnahme ist freiwillig. Sie können sich darauf verlassen, dass ihre Angaben anonym erhoben werden.

Bei Fragen oder sonstigen Anmerkungen können sie sich gerne per E-Mail bei robert.staeck@uzh.ch oder stefan.albisser@uzh.ch melden.

Nochmals vielen Dank für Ihre Mithilfe!

○ Ich stimme zu, dass meine Daten gemäss den hier aufgeführten Angaben verarbeitet werden. Ich bin momentan in der Psychotherapieausbildung.

○ Ich möchte nicht teilnehmen.

1 aktive(r) Filter

Filter IN02/F1
Wenn eine der folgenden Antwortoption(en) ausgewählt wurde: **2, -9**
Dann nach dem Klick auf "Weiter" den Text **X101** anzeigen und das Interview beenden

Seite 02

SD01

1. Alter

- ◯ 20-24
- ◯ 25-29
- ◯ 30-34
- ◯ 35-39
- ◯ 40-44
- ◯ 45-49
- ◯ 50-54
- ◯ 55-59
- ◯ 60+

SD02

2. Geschlecht

- ◯ weiblich
- ◯ männlich
- ◯ divers

SD07

3. Wie sieht Ihr bisheriger Ausbildungsweg aus?

- ☐ Ich habe ein Psychologiestudium absolviert.
- ☐ Ich habe ein Medizinstudium absolviert.

SD03

4. Therapierichtung, gemäss Abschluss (bei Mischformen auch mehrere Antworten möglich)

- ☐ Humanistische Psychotherapie
- ☐ Systemische Psychotherapie
- ☐ Tiefenpsychologie / Psychoanalyse
- ☐ Verhaltenstherapie (kognitiv / kognitiv-behavioraler Ansatz)
- ☐ andere: ______

SD04

5. Ausbildungsland (Psychotherapieausbildung)

- ◯ Schweiz
- ◯ Deutschland

3 aktive(r) Filter

Filter SD04/F1
Wenn eine der folgenden Antwortoption(en) ausgewählt wurde: **1**
Dann Seite(n) **jump1** des Fragebogens anzeigen (sonst ausblenden)

Filter SD04/F2
Wenn eine der folgenden Antwortoption(en) ausgewählt wurde: **2**
Dann Seite(n) **jump2** des Fragebogens anzeigen (sonst ausblenden)

Filter SD04/F3

Wenn eine der folgenden Antwortoption(en) ausgewählt wurde: **-9**
Dann nach dem Klick auf "Weiter" direkt zur Seite **%page%** springen

---

Seite 03

jump1

SD05

6. Stand der Therapieausbildung

Wie weit fortgeschritten ist Ihre Ausbildung bereits in den verschiedenen Bereichen?

	0%	100%
Theoretischer Teil		
Praktischer Teil		
Selbsterfahrung		
Supervision		

Seite 04

jump2

SD06

7. Stand der Therapieausbildung

Wie weit fortgeschritten ist Ihre Ausbildung bereits in den verschiedenen Bereichen?

	0%	100%
Theoretische Ausbildung		
Praktische Ausbildung		
Praktische Tätigkeit I		
Praktische Tätigkeit II		
Selbsterfahrung		

Seite 05

jump3

W103

Im ersten Schritt soll der Begriff **E-Health** sowie verschiedene **E-Health-Bereiche** genauer beschrieben werden. Nach einer kurzen Definition können Sie jeweils angeben, wie viel Sie über die bestimmten Bereiche bereits wissen.

W101

E-Mental-Health Allgemein

E-Mental-Health ist ein allgemeiner Begriff für Informations- und Kommunikations-Technologien (sowohl Geräte wie z.B. Mobiltelefone & Computer als auch Programme wie z.B. Apps). Diese können unterstützend wirken und helfen, die psychische Gesundheit einer Person zu verbessern. E-Mental-Health-Technologien können sehr unterschiedlich sein.

W102

8. Was wissen Sie bereits über E-Mental-Health?

	Stimme gar nicht zu	Stimme eher nicht zu	Weder noch	Stimme eher zu	Stimme voll zu
Darunter kann ich mir schon etwas vorstellen.	○	○	○	○	○
Ich kann mir vorstellen, was mich beim Einsatz von E-Mental-Health als therapeutisches Mittel erwartet.	○	○	○	○	○
Ich verfüge bereits über Wissen bezüglich E-Mental-Health.	○	○	○	○	○

Seite 06

W201

Psychotherapie via Telefon

Unter dem Überbegriff «Telehealth» versteht man die *Anwendung von Technologieformen* wie Telefon, E-Mail, Web-Foren oder Kommunikation via Video-Konferenz *im Gesundheitskontext*.

Eine **Psychotherapie** kann anstelle einer Face-to-Face-Therapie auch **per Telefon** durchgeführt werden. Dadurch kann der Zugang zu einer Psychotherapie für bestimmte *Personengruppen* (z.B. Personen mit spezifischen Beeinträchtigungen, Personen wohnhaft in ländlichen Gebieten,...) sowie in *ausserordentlichen Zuständen* (z.B. Corona-Krise) erleichtert werden.

W202

9. Was wissen Sie bereits über Psychotherapie via Telefon?

	Stimme gar nicht zu	Stimme eher nicht zu	Weder noch	Stimme eher zu	Stimme voll zu
Darunter kann ich mir schon etwas vorstellen.	○	○	○	○	○
Ich kann mir vorstellen, was mich beim Einsatz von „Psychotherapie via Telefon“ als therapeutisches Mittel erwartet.	○	○	○	○	○
Ich verfüge bereits über Wissen bezüglich „Psychotherapie via Telefon“.	○	○	○	○	○

Seite 07

W301

Psychotherapie via Video-Konferenz

Unter dem Überbegriff «Telehealth» versteht man die *Anwendung von Technologieformen* wie Telefon, E-Mail, Web-Foren oder Kommunikation via Video-Konferenz *im Gesundheitskontext*.

Eine **Psychotherapie** kann anstelle einer Face-to-Face-Therapie auch **per Videokonferenz (z.B. per Skype)** durchgeführt werden. Dadurch kann der Zugang zu einer Psychotherapie für bestimmte *Personengruppen* (z.B. Personen mit spezifischen Beeinträchtigungen, Personen wohnhaft in ländlichen Gebieten,...) sowie in *ausserordentlichen Zuständen* (z.B. Corona-Krise) erleichtert werden. Das Ziel ist es, durch sowohl auditive als auch visuelle Reize einer klassischen Psychotherapie möglichst nahe zu kommen.

W302

10. Was wissen Sie bereits über Psychotherapie via Videokonferenz?

	Stimme gar nicht zu	Stimme eher nicht zu	Weder noch	Stimme eher zu	Stimme voll zu
Darunter kann ich mir schon etwas vorstellen.	○	○	○	○	○
Ich kann mir vorstellen, was mich beim Einsatz von „Psychotherapie via Videokonferenz“ als therapeutisches Mittel erwartet.	○	○	○	○	○
Ich verfüge bereits über Wissen bezüglich „Psychotherapie via Videokonferenz“.	○	○	○	○	○

Seite 08

W401

Virtual Reality

Virtuelle Realität (VR) bezeichnet Computersimulationen, die für den Nutzer die Illusion erzeugen, tatsächlich in der virtuellen Welt anwesend zu sein. VR erlaubt eine Interaktion zwischen der Person und dem Stimulus. Dies kann zu **realitätsnahen und emotionalen Konfrontationen mit dem präsentierten Stimulus** führen.

Momentan wird VR vor allem in der Therapie für Angststörungen angewendet, genauer in der Expositionstherapie. Das Angst auslösende Element (z.B. Höhe, Spinne,...) kann damit der Person nähergebracht werden. Die Hemmschwelle ist bei VR-Reizen oft tiefer als bei einer realen Konfrontation.

W402

11. Was wissen Sie bereits über Virtual Reality?

	Stimme gar nicht zu	Stimme eher nicht zu	Weder noch	Stimme eher zu	Stimme voll zu
Darunter kann ich mir schon etwas vorstellen.	○	○	○	○	○
Ich kann mir vorstellen, was mich beim Einsatz von „Virtual Reality“ als therapeutisches Mittel erwartet.	○	○	○	○	○
Ich verfüge bereits über Wissen bezüglich „Virtual Reality“.	○	○	○	○	○

W501

Internet-Programme ohne therapeutische Assistenz (nicht geleitete Programme)

Einige **internetbasierte Programme** nutzen Verfahren, welche in der Face-to-Face Therapie bereits erfolgreich waren. Online Programme folgen oft einer ähnlichen Struktur wie eine traditionelle Therapie. Unter anderem helfen spezifische Instruktionen, Symptome zu reduzieren. Beispielsweise kann ein solches Programm Personen beibringen, wie diese ihre negativen Gedanken los werden können.

Die Programme können als reines Selbsthilfeprogramm oder mit zusätzlicher therapeutischer Unterstützung durchgeführt werden. In diesem Abschnitt handelt es sich um ersteres, also **Programme, welche nicht psychotherapeutisch begleitet** werden.

Da Sie als Therapeut*in diese Art von Therapie nicht selbst durchführen, beziehen sich die später folgenden Fragen darauf, ob Sie **solche Programme ihren Patient*innen <u>empfehlen</u>** würden.

W502

12. Was wissen Sie bereits über nicht geleitete Programmen?

	Stimme gar nicht zu	Stimme eher nicht zu	Weder noch	Stimme eher zu	Stimme voll zu
Darunter kann ich mir schon etwas vorstellen.	○	○	○	○	○
Ich kann mir vorstellen, was mich beim Einsatz von „nicht geleiteten Programmen“ als therapeutisches Mittel erwartet.	○	○	○	○	○
Ich verfüge bereits über Wissen bezüglich „nicht geleiteten Programmen“.	○	○	○	○	○

W601

Internet-Programme mit therapeutischer Assistenz (geleitete Programme)

Als Therapeut*in können Sie Ihre Patient*innen in der Nutzung von internetbasierten Programmen unterstützen. Diese Unterstützung kann unterschiedliche Formen annehmen: Feedback geben, Fragen beantworten, als Erinnerungsstütze wirken, usw.

Dazu können verschiedene Hilfsmittel (Telefon, E-Mail, Video-Konferenz, …) genutzt werden. Die Intensität des Kontaktes mit der therapierenden Person kann hierbei variieren.

W602

13. Was wissen Sie bereits über geleitete Programme?

	Stimme gar nicht zu	Stimme eher nicht zu	Weder noch	Stimme eher zu	Stimme voll zu
Darunter kann ich mir schon etwas vorstellen.	○	○	○	○	○
Ich kann mir vorstellen, was mich beim Einsatz von „geleiteten Programmen“ als therapeutisches Mittel erwartet.	○	○	○	○	○
Ich verfüge bereits über Wissen bezüglich „geleiteten Programmen“.	○	○	○	○	○

U109

Was halten Sie von der Verwendung von folgenden Programmen für die Behandlung psychisch erkrankter Personen?

U101

14. Folgende Programme wären eine wertvolle Erweiterung der bisherigen Behandlungsmassnahmen.

	Stimme gar nicht zu	Stimme eher nicht zu	Weder noch	Stimme eher zu	Stimme voll zu
E-Mental-Health Allgemein	○	○	○	○	○
Psychotherapie via Telefon	○	○	○	○	○
Psychotherapie via Videkonferenz	○	○	○	○	○
Virtual Reality	○	○	○	○	○
Nicht geleitete Programme	○	○	○	○	○
Geleitete Programme	○	○	○	○	○

U102

15. Meine Arbeitskolleg*innen würden den Einsatz von folgenden Programmen befürworten.

	Stimme gar nicht zu	Stimme eher nicht zu	Weder noch	Stimme eher zu	Stimme voll zu
E-Mental-Health Allgemein	○	○	○	○	○
Psychotherapie via Telefon	○	○	○	○	○
Psychotherapie via Videokonferenz	○	○	○	○	○
Virtual Reality	○	○	○	○	○
Nicht geleitete Programme	○	○	○	○	○
Geleitete Programme	○	○	○	○	○

U103

16. Die technische Ausstattung meines beruflichen Umfeldes ist ausreichend für die Anwendung von folgenden Programmen.

	Stimme gar nicht zu	Stimme eher nicht zu	Weder noch	Stimme eher zu	Stimme voll zu
E-Mental-Health Allgemein	○	○	○	○	○
Psychotherapie via Telefon	○	○	○	○	○
Psychotherapie via Videkonferenz	○	○	○	○	○
Virtual Reality	○	○	○	○	○
Nicht geleitete Programme	○	○	○	○	○
Geleitete Programme	○	○	○	○	○

U104

17. Die Patient*innen erwarten von uns den Einsatz folgender Programme.

	Stimme gar nicht zu	Stimme eher nicht zu	Weder noch	Stimme eher zu	Stimme voll zu
E-Mental-Health Allgemein	○	○	○	○	○
Psychotherapie via Telefon	○	○	○	○	○
Psychotherapie via Videokonferenz	○	○	○	○	○
Virtual Reality	○	○	○	○	○
Nicht geleitete Programme	○	○	○	○	○
Geleitete Programme	○	○	○	○	○

U105

18. Ich hätte die nötigen technischen Kenntnisse für den Umgang mit folgenden Programmen.

	Stimme gar nicht zu	Stimme eher nicht zu	Weder noch	Stimme eher zu	Stimme voll zu
E-Mental-Health Allgemein	○	○	○	○	○
Psychotherapie via Telefon	○	○	○	○	○
Psychotherapie via Videkonferenz	○	○	○	○	○
Virtual Reality	○	○	○	○	○
Nicht geleitete Programme	○	○	○	○	○
Geleitete Programme	○	○	○	○	○

U106

19. Ich könnte den Umgang mit folgenden Programmen vermutlich schnell lernen.

	Stimme gar nicht zu	Stimme eher nicht zu	Weder noch	Stimme eher zu	Stimme voll zu
E-Mental-Health Allgemein	○	○	○	○	○
Psychotherapie via Telefon	○	○	○	○	○
Psychotherapie via Videkonferenz	○	○	○	○	○
Virtual Reality	○	○	○	○	○
Nicht geleitete Programme	○	○	○	○	○
Geleitete Programme	○	○	○	○	○

U107

20. Die Bedienung von folgenden Programmen wäre für mich einfach.

	Stimme gar nicht zu	Stimme eher nicht zu	Weder noch	Stimme eher zu	Stimme voll zu
E-Mental-Health Allgemein	○	○	○	○	○
Psychotherapie via Telefon	○	○	○	○	○
Psychotherapie via Videkonferenz	○	○	○	○	○
Virtual Reality	○	○	○	○	○
Nicht geleitete Programme	○	○	○	○	○
Geleitete Programme	○	○	○	○	○

U108

21. Folgende Programme könnten die Gesundheit der Patient*innen zusätzlich verbessern.

	Stimme gar nicht zu	Stimme eher nicht zu	Weder noch	Stimme eher zu	Stimme voll zu
E-Mental-Health Allgemein	○	○	○	○	○
Psychotherapie via Telefon	○	○	○	○	○
Psychotherapie via Videkonferenz	○	○	○	○	○
Virtual Reality	○	○	○	○	○
Nicht geleitete Programme	○	○	○	○	○
Geleitete Programme	○	○	○	○	○

Seite 12

Z101

22. Welche Programme würden Sie in der Prävention einsetzen?

	Stimme gar nicht zu	Stimme eher nicht zu	Weder noch	Stimme eher zu	Stimme voll zu
E-Mental-Health Allgemein	○	○	○	○	○
Psychotherapie via Telefon	○	○	○	○	○
Psychotherapie via Videkonferenz	○	○	○	○	○
Virtual Reality	○	○	○	○	○
Nicht geleitete Programme	○	○	○	○	○
Geleitete Programme	○	○	○	○	○

Z102

23. Welche Programme würden Sie in der Akutphase als Ergänzung zur Therapie einsetzen?

	Stimme gar nicht zu	Stimme eher nicht zu	Weder noch	Stimme eher zu	Stimme voll zu
E-Mental-Health Allgemein	○	○	○	○	○
Psychotherapie via Telefon	○	○	○	○	○
Psychotherapie via Videokonferenz	○	○	○	○	○
Virtual Reality	○	○	○	○	○
Nicht geleitete Programme	○	○	○	○	○
Geleitete Programme	○	○	○	○	○

Z103

24. Welche Programme würden Sie in der Akutphase als Ersatz zur Therapie einsetzen?

	Stimme gar nicht zu	Stimme eher nicht zu	Weder noch	Stimme eher zu	Stimme voll zu
E-Mental-Health Allgemein	○	○	○	○	○
Psychotherapie via Telefon	○	○	○	○	○
Psychotherapie via Videokonferenz	○	○	○	○	○
Virtual Reality	○	○	○	○	○
Nicht geleitete Programme	○	○	○	○	○
Geleitete Programme	○	○	○	○	○

Z104

25. Welche Programme würden Sie in der Nachsorge einsetzen?

	Stimme gar nicht zu	Stimme eher nicht zu	Weder noch	Stimme eher zu	Stimme voll zu
E-Mental-Health Allgemein	○	○	○	○	○
Psychotherapie via Telefon	○	○	○	○	○
Psychotherapie via Videokonferenz	○	○	○	○	○
Virtual Reality	○	○	○	○	○
Nicht geleitete Programme	○	○	○	○	○
Geleitete Programme	○	○	○	○	○

Z107

26. Blended Treatments

Mit Blended Treatments sind psychotherapeutische Behandlungen gemeint, die teilweise im traditionellen Face-to-Face-Setting durchgeführt werden und durch internetbasierte Interventionen ergänzt werden. Was ist aus Ihrer Sicht der optimale Mix zwischen herkömmlichen und Online-Angeboten?

Ein Wert in der Mitte meint, dass sie einen Mix aus 50% herkömmlichen Angeboten und 50% Online-Angeboten als optimal betrachten.

	100 % Herkömmliche Angebote	100 % Online-Angebote
E-Mental-Health Allgemein		
Psychotherapie via Telefon		
Psychotherapie via Video-Konferenz		
Virtual Reality		
Nicht geleitete Programme		
Geleitete Programme		

VN01

27. Bei den folgenden Programmen habe ich Bedenken bezüglich der Datensicherheit.

	Stimme gar nicht zu	Stimme eher nicht zu	Weder noch	Stimme eher zu	Stimme voll zu
E-Mental-Health Allgemein	○	○	○	○	○
Psychotherapie via Telefon	○	○	○	○	○
Psychotherapie via Videokonferenz	○	○	○	○	○
Virtual Reality	○	○	○	○	○
Nicht geleitete Programme	○	○	○	○	○
Geleitete Programme	○	○	○	○	○

VN02

28. Die folgenden Programme finde ich zu unpersönlich und zu wenig interaktiv.

	Stimme gar nicht zu	Stimme eher nicht zu	Weder noch	Stimme eher zu	Stimme voll zu
E-Mental-Health Allgemein	○	○	○	○	○
Psychotherapie via Telefon	○	○	○	○	○
Psychotherapie via Videokonferenz	○	○	○	○	○
Virtual Reality	○	○	○	○	○
Nicht geleitete Programme	○	○	○	○	○
Geleitete Programme	○	○	○	○	○

VN03

29. Bei den folgenden Programmen habe ich Sorgen bezüglich meiner Verantwortung als Psychotherapeut*in gegenüber meinen Patienten*innen (zum Beispiel in Notsituationen).

	Stimme gar nicht zu	Stimme eher nicht zu	Weder noch	Stimme eher zu	Stimme voll zu
E-Mental-Health Allgemein	○	○	○	○	○
Psychotherapie via Telefon	○	○	○	○	○
Psychotherapie via Videokonferenz	○	○	○	○	○
Virtual Reality	○	○	○	○	○
Nicht geleitete Programme	○	○	○	○	○
Geleitete Programme	○	○	○	○	○

VN04

30. Bei den folgenden Programmen habe ich rechtliche Bedenken (zum Beispiel da ich nicht genügend informiert bin über die aktuelle Gesetzteslage).

	Stimme gar nicht zu	Stimme eher nicht zu	Weder noch	Stimme eher zu	Stimme voll zu
E-Mental-Health Allgemein	○	○	○	○	○
Psychotherapie via Telefon	○	○	○	○	○
Psychotherapie via Videokonferenz	○	○	○	○	○
Virtual Reality	○	○	○	○	○
Nicht geleitete Programme	○	○	○	○	○
Geleitete Programme	○	○	○	○	○

VN05

31. Bei den folgenden Programmen habe ich Sorgen, dass ich keine tragfähige therapeutische Beziehung aufbauen kann.

	Stimme gar nicht zu	Stimme eher nicht zu	Weder noch	Stimme eher zu	Stimme voll zu
E-Mental-Health Allgemein	○	○	○	○	○
Psychotherapie via Telefon	○	○	○	○	○
Psychotherapie via Videokonferenz	○	○	○	○	○
Virtual Reality	○	○	○	○	○
Nicht geleitete Programme	○	○	○	○	○
Geleitete Programme	○	○	○	○	○

VN06

32. Bei den folgenden Programmen denke ich, dass es die Vermittlung von Informationen und Übungen vereinfacht.

	Stimme gar nicht zu	Stimme eher nicht zu	Weder noch	Stimme eher zu	Stimme voll zu
E-Mental-Health Allgemein	○	○	○	○	○
Psychotherapie via Telefon	○	○	○	○	○
Psychotherapie via Videokonferenz	○	○	○	○	○
Virtual Reality	○	○	○	○	○
Nicht geleitete Programme	○	○	○	○	○
Geleitete Programme	○	○	○	○	○

VN07

33. Bei den folgenden Programmen finde ich es vorteilhaft, da es für Patient*innen flexibel verfügbar wäre.

	Stimme gar nicht zu	Stimme eher nicht zu	Weder noch	Stimme eher zu	Stimme voll zu
E-Mental-Health Allgemein	○	○	○	○	○
Psychotherapie via Telefon	○	○	○	○	○
Psychotherapie via Videokonferenz	○	○	○	○	○
Virtual Reality	○	○	○	○	○
Nicht geleitete Programme	○	○	○	○	○
Geleitete Programme	○	○	○	○	○

VN08

34. Bei den folgenden Programmen halte ich es für Personen in ländlichen Regionen besonders hilfreich, da es den Zugang vereinfacht.

	Stimme gar nicht zu	Stimme eher nicht zu	Weder noch	Stimme eher zu	Stimme voll zu
E-Mental-Health Allgemein	○	○	○	○	○
Psychotherapie via Telefon	○	○	○	○	○
Psychotherapie via Videokonferenz	○	○	○	○	○
Virtual Reality	○	○	○	○	○
Nicht geleitete Programme	○	○	○	○	○
Geleitete Programme	○	○	○	○	○

VN09

35. Bei den folgenden Programmen wäre es einfacher als bisher, mit Patient*innen im Kontakt zu bleiben.

	Stimme gar nicht zu	Stimme eher nicht zu	Weder noch	Stimme eher zu	Stimme voll zu
E-Mental-Health Allgemein	○	○	○	○	○
Psychotherapie via Telefon	○	○	○	○	○
Psychotherapie via Videokonferenz	○	○	○	○	○
Virtual Reality	○	○	○	○	○
Nicht geleitete Programme	○	○	○	○	○
Geleitete Programme	○	○	○	○	○

ER09

36. Die folgende Einschätzung bezieht sich auf den Zeitraum zwischen Anfangs März 2020 und heute.

Zu wie viel Prozent benutzten Sie die jeweiligen E-Health-Bereiche in Ihrem therapeutischen Arbeiten?

- für Psychotherapie: Wie viel Prozent Ihrer Psychotherapie führten Sie durch das Telefon bzw. durch Videotelefonie durch?

- für VR: Wie viele Patient*innen betreuen Sie mithilfe von Virtual-Reality?

- Für (nicht) geleitete Programme: Wie viel Prozent ihrer Patient*innnen betreuten Sie mitheilfe eines E-Health-Programms (bzw. empfehlten sie lediglich ein solches Programm)?

nie	immer	
		Psychotherapie via Telefon
		Psychotherapie via Videokonferenz
		Virtual Reality
		Nicht geleitete Programme
		Geleitete Programme

ER08

37. Wie waren Ihre Erfahrungen mit den folgenden Programmen?

	negativ	positiv	keine Erfahrung
E-Mental Health Allgemein			
Psychotherapie via Telefon			
Psychotherapie via Video-Konferenz			
Virtual Reality			
Nicht geleitete Programme			
Geleitete Programme			

AZ01

38. Ich könnte mir vorstellen, solche Programme in meine Tätigkeit einzubinden.

	Stimme gar nicht zu	Stimme eher nicht zu	Weder noch	Stimme eher zu	Stimme voll zu
E-Mental-Health Allgemein	○	○	○	○	○
Psychotherapie via Telefon	○	○	○	○	○
Psychotherapie via Videokonferenz	○	○	○	○	○
Virtual Reality	○	○	○	○	○
Nicht geleitete Programme	○	○	○	○	○
Geleitete Programme	○	○	○	○	○

AZ02

39. Ich habe mir vorgenommen, im nächsten Jahr solche Programme in meiner Tätigkeit auszuprobieren.

	Stimme gar nicht zu	Stimme eher nicht zu	Weder noch	Stimme eher zu	Stimme voll zu
E-Mental-Health Allgemein	○	○	○	○	○
Psychotherapie via Telefon	○	○	○	○	○
Psychotherapie via Videokonferenz	○	○	○	○	○
Virtual Reality	○	○	○	○	○
Nicht geleitete Programme	○	○	○	○	○
Geleitete Programme	○	○	○	○	○

AZ03

40. Wie hoch ist ihre Intention, folgende Programme in Ihrer Tätigkeit jemals zu gebrauchen?

	keine Intention	sehr starke Intention
E-Mental-Health Allgemein		
Psychotherapie via Telefon		
Psychotherapie via Videokonferenz		
Virtual Reality		
Nicht geleitete Programme		
Geleitete Programme		

CV01

Die Corona-Krise hat unser aller Leben mehr oder weniger beeinflusst. Auch viele Psychotherapeut*innen waren in dieser Zeit gefordert und viele entschieden sich oder waren zum Teil sogar gezwungen, die Therapien mit Patientinnen und Patienten unkonventionell durchzuführen.

Wir würden Sie für die folgenden Fragen bitten, sich **an die Zeit vor Corona zurückzuerinnern**. Gehen Sie gedanklich zurück in den **Herbst 2019** und probieren Sie die *folgenden Fragen so zu beantworten, wie sie das damals gemacht hätten*.

Wir bitten Sie diese Fragen lediglich für den Bereich "E-Mental-Health Allgemein" zu beantworten. Im Folgenden beschreiben wir nochmals, was darunter zu verstehen ist.

CV02

E-Mental-Health Allgemein

E-Mental-Health ist ein allgemeiner Begriff für **Informations- und Kommunikations-Technologien** (sowohl *Geräte* wie z.B. Mobiltelefone & Computer als auch *Programme* wie z.B. Apps). Diese können unterstützend wirken und helfen, die psychische Gesundheit einer Person zu verbessern. E-Mental-Health Technologien können sehr unterschiedlich sein.

CV06

41. Was wussten Sie im Herbst 2019 (!) bereits über E-Mental-Health?

	Stimme gar nicht zu	Stimme eher nicht zu	Weder noch	Stimme eher zu	Stimme voll zu
Darunter konnte ich mir schon etwas vorstellen.	○	○	○	○	○
Ich konnte mir vorstellen, was mich beim Einsatz von E-Mental-Health als therapeutisches Mittel erwartet.	○	○	○	○	○
Ich verfügte bereits über Wissen bezüglich E-Mental-Health.	○	○	○	○	○

CV03

42. Was hielten Sie im Herbst 2019 (!) von der Verwendung von E-Mental-Health für die Behandlung von Personen mit einer psychischen Erkrankung?

	Stimme nicht zu	Stimme eher nicht zu	Weder noch	Stimme eher zu	Stimme voll zu
E-Mental-Health wäre eine wertvolle Erweiterung der bisherigen Behandlungsmassnahmen.	○	○	○	○	○
Meine Kolleg*innen hätten den Einsatz von E-Mental-Health befürwortet.	○	○	○	○	○
Die technische Ausstattung meines beruflichen Umfeldes wäre für die Anwendung von E-Mental-Health ausreichend gewesen.	○	○	○	○	○
Die Patient*innen erwarteten von uns den Einsatz von E-Mental-Health.	○	○	○	○	○
Ich hätte die nötigen technischen Kenntnisse für den Umgang mit E-Mental-Health gehabt.	○	○	○	○	○
Ich hätte den Umgang mit E-Mental-Health vermutlich schnell lernen können.	○	○	○	○	○
Die Bedienung von E-Mental-Health wäre für mich einfach gewesen.	○	○	○	○	○
E-Mental-Health könnte die Gesundheit der Patient*innen zusätzlich verbessern.	○	○	○	○	○

CV04

43. Wann hätten Sie sich im Herbst 2019 (!) vorstellen können E-Mental-Health einzusetzen?

	Stimme gar nicht zu	Stimme eher nicht zu	Weder noch	Stimme eher zu	Stimme voll zu
In der Prävention	○	○	○	○	○
In der Akutphase als Ergänzung zur Therapie	○	○	○	○	○
In der Akutphase als Ersatz zur Therapie	○	○	○	○	○
In der Nachversorgung	○	○	○	○	○

CV05

44. Was waren aus Ihrer Sicht im Herbst 2019 (!) Vor- und Nachteile von E-Mental-Health im Vergleich zu herkömmlichen Angeboten?

**Was E-Mental Health angeht …**	Stimme gar nicht zu	Stimme eher nicht zu	Weder noch	Stimme eher zu	Stimme voll zu
... hatte ich Bedenken bezüglich der Datensicherheit.	○	○	○	○	○
... fand ich es zu unpersönlich und zu wenig interaktiv.	○	○	○	○	○
... hatte ich Sorgen bezüglich meiner Verantwortung als Psychotherapeut*in gegenüber meinen Patienten*innen (z.B. in Notsituationen).	○	○	○	○	○
... hatte ich rechtliche Bedenken (z.B. da ich nicht genügend informiert bin über die aktuelle Gesetzteslage).	○	○	○	○	○
... hatte ich Sorgen, dass ich keine so tragfähige therapeutische Beziehung aufbauen kann.	○	○	○	○	○
... dachte ich, dass es die Vermittlung von Informationen und Übungen vereinfacht.	○	○	○	○	○
...fand ich es vorteilhaft, da es für Patient*innen flexibel verfügbar wäre.	○	○	○	○	○
...hielt ich es für Personen in ländlichen Regionen besonders hilfreich, da es den Zugang vereinfacht.	○	○	○	○	○
...wäre es einfacher gewesen als bis anhin, mit Patient*innen im Kontakt zu bleiben.	○	○	○	○	○

CV07

45. Was war ihre Intention für die Zukunft (im Herbst 2019!)?

	Stimme gar nicht zu	Stimme eher nicht zu	Weder noch	Stimme eher zu	Stimme voll zu
Ich konnte mir vorstellen, E-Mental-Health in meine Tätigkeit einzubinden.	○	○	○	○	○
Ich hatte mir vorgenommen, im folgenden Jahr E-Mental-Health in meiner Tätigkeit auszuprobieren.	○	○	○	○	○

CV08

nicht vorhanden sehr hoch

Wie stark war Ihre Intention E-Mental-Health in ihrer Tätigkeit jemals zu gebrauchen (im Herbst 2019!)?

CV09

46. Zu wie viel Prozent benutzten Sie E-Health in Ihrem therapeutischen Arbeiten?

Bei wieviel Prozent Ihrerer Patient*innen benutz(t)en Sie eine Art von E-Health (z.B. bei 1 von 10 = 10%)

E-Health allgemein (Herbst 2019)

E-Health allgemein (aktuell, seit März 2020)

CV11

47. Wie waren Ihre Erfahrungen mit den folgenden Programmen?

Negativ Positiv

keine Erfahrung

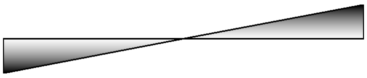

E-Health allgemein (Herbst 2019)

E-Health allgemein (aktuell, seit März 2020)

Seite 18

CO01

Die Corona Pandemie hat im Jahr 2020 unser aller Leben beeinflusst. Psychotherapeut*innen waren in dieser Zeit gefordert und viele führten die Behandlungen auf unkonventionellere Weise durch als gewohnt.

Welche Erfahrungen haben Sie als Therapeut*innen in dieser Zeit mit E-Health gemacht?

Allgemein

Positiv

Negativ

Seite 19

CV10

48. Wie stark würden Sie die wissenschaftliche Evidenz bezüglich Wirksamkeit der unterschiedlichen E-Health-Bereiche einschätzen?

	Sehr gering	Sehr hoch
E-Mental Health allgemein (aktuell, seit März 2020)		
E-Mental Health allgemein (Herbst 2019)		
Psychotherapie via Telefon		
Psychotherapie via Video-Konferenz		
Virtual Reality		
nicht geleitete Programme		
geleitete Programme		

Seite 20

X102

49. Haben Sie gewisse E-Mental-Health Anwendungen vermisst oder gibt es sonstige offene Punkte? Dann teilen Sie uns diese mit.

Seite 21

X103

50. Vielen Dank für Ihre Teilnahme und Zeit! Als Dankeschön verlosen wir vier Migros-Gutscheine im Wert von insgesamt 250 Franken (1x 100 Fr. und 3x 50 Fr). Falls Sie kein Interesse daran haben, klicken Sie auf weiter, um den Fragebogen abzuschliessen.

- ☐ Ich will am Gewinnspiel für Migros-Gutscheine teilnehmen. Ich willige ein, dass meine E-Mail-Adresse bis zur Ziehung der Gewinner gespeichert wird. Meine Angaben in dieser Befragung bleiben weiterhin anonym. Meine E-Mail-Adresse wird nicht an Dritte weitergegeben.
- ☐ Ich interessiere mich für die Ergebnisse dieser Studie und hätte gerne eine Zusammenfassung per E-Mail, sobald diese fertig ausgewertet sind.

Vielen Dank für Ihre Teilnahme!

Wir möchten uns ganz herzlich für Ihre Mithilfe bedanken.

Bei Fragen oder sonstigen Anmerkungen können sie sich gerne per E-Mail bei robert.staeck@uzh.ch oder stefan.albisser@uzh.ch melden.

Ihre Antworten wurden gespeichert, Sie können das Browser-Fenster nun schließen.

Möchten Sie in Zukunft an interessanten und spannenden Online-Befragungen teilnehmen?

Wir würden uns sehr freuen, wenn Sie Ihre E-Mail-Adresse für das SoSci Panel anmelden und damit wissenschaftliche Forschungsprojekte unterstützen.

E-Mail: ____________ [Am Panel teilnehmen]

Die Teilnahme am SoSci Panel ist freiwillig, unverbindlich und kann jederzeit widerrufen werden. Das SoSci Panel speichert Ihre E-Mail-Adresse nicht ohne Ihr Einverständnis, sendet Ihnen keine Werbung und gibt Ihre E-Mail-Adresse nicht an Dritte weiter.

Sie können das Browserfenster selbstverständlich auch schließen, ohne am SoSci Panel teilzunehmen.

Robert Staeck, Universität Zürich – 2020
