# Supplementary material for: Acceptance of E-Mental Health Services for Different Application Purposes Among Psychotherapists in Clinical Training in Germany and Switzerland: Secondary Analysis of a Cross-Sectional Survey
Source: Front Digit Health. 2022 Feb 28;4:840869. doi: 10.3389/fdgth.2022.840869 (PMC8918841; doi:10.3389/fdgth.2022.840869)
Supplement: Supplementary file 2 [file Data_Sheet_2.PDF]

Translation and Source of Items

Construct	Items (rating scale)	Source
Acceptance	I could imagine including the following EMH services in my work. (1-5)*<br>I intend to try out the following EMH service in my work within the next year. (1-5)*<br>How high is your intention to use the following EMH service in your work ever? (0-100)**	Adapted from Hennemann et al. 2017, Venkatesh et al. 2003; Elfeddali et al. 2013
UTAUT predictors	*Performance expectancy (1-5)*:*<br>The following EMH service would be a useful extension to existing treatment measures.<br>The following EMH services could improve patient's health status.<br>*Effort expectancy (1-5)*:*<br>I would find the following EMH service easy to use and to understand.<br>I would easily learn to operate the following EMH service.<br>*Social influence (1-5)*:*<br>My colleagues would approve the use of the following EMH service.<br>Our patients endorse the use of the following EMH services.<br>*Facilitating conditions (1-5)*:*<br>I have the necessary technical preconditions for using the following EMH services.<br>The technical equipment of my professional environment is adequate for the implementation of EMH services.	Adapted from Hennemann et al. 2017; Venkatesh et al. 2003
Application purposes	Which of the following EMH services would you use for prevention? (1-5)*<br>Which of the following EMH services would you use for aftercare? (1-5)*<br>Which of the following EMH services would you use as an addition to therapy? (1-5)*<br>Which of the following EMH services would you use as a substitute for therapy? (1-5)*	Self-constructed
Advantages	I believe the following EMH services would simplify the provision of information and exercises. (1-5)*<br>Regarding the following EMH services, I think it is beneficial that they are flexibly accessible. (1-5)*<br>The following EMH services are especially helpful for patients living in rural areas as they facilitate the access. (1-5)*	Adapted from Hennemann et al. 2016; Bendelin et al. 2011; Berger 2015; Wells et al. 2007

Translation and Source of Items

Construct	Items (rating scale)	Source
	The following EMH services allow me to maintain contact with my patients. (1-5)*	
Barriers	Regarding the following EMH services, I have concerns about data security. (1-5)*<br>The following EMH services are too impersonal and not interactive enough. (1-5)*<br>Regarding the following EMH services, I have concerns about my responsibility as a psychotherapist towards my patients (e.g., in emergency situations). (1-5)*<br>Regarding the following EMH services, I have legal concerns (e.g., because I am not sufficiently informed about the current legal situation). (1-5)*<br>Regarding the following EMH services, I have concerns about not being able to build a viable therapeutic relationship. (1-5)*	Adapted from Hennemann et al. 2016; Bendelin et al. 2011; Berger 2015; Wells et al. 2007
Knowledge about EMH	I have an idea of what the following EMH services are. (1-5)*<br>I know what I can expect when using the following EMH services as a therapeutic tool. (1-5)*<br>I have some knowledge on the following EMH services. (1-5)*	Adapted from Hennemann et al. 2017; Ebert et al., 2015
EMH experience	In percentage, how much do you already use the following EMH services in your therapeutic work? (0-100)***<br>- for psychotherapy: how much percentage of your psychotherapy sessions did you execute by phone or videoconference, respectively?<br>- for VR: How many patients do you serve with VR?<br>- for (un-)guided programs: how much percentage of your patients do you serve with e-health programs? (or did you recommend such a program?)	Adapted from Hennemann et al. 2017; Eichenberg et al. 2013
Evidence assessment	How strong would you rate the scientific evidence base of the following EMH services (0-100)****	Self-constructed

*rating scale: (1) *totally disagree* to (5) *totally agree*
**rating scale: (1) *no intention* to (100) *very strong intention*
***rating scale: (1) *never* to (100) *always*
****rating scale: (1) *very low* to (100) *very high*
